# Study of Ammonia Adsorption on Magnetite Surfaces with Molecular Dynamics Simulations

**DOI:** 10.3390/molecules29143276

**Published:** 2024-07-11

**Authors:** Nikoleta Ivanova, Vasil Karastoyanov, Iva Betova, Martin Bojinov

**Affiliations:** 1 Department of Physical Chemistry, University of Chemical Technology and Metallurgy, 8 Kliment Ohridski Blvd., 1756 Sofia, Bulgaria; n.ivanova@uctm.edu (N.I.); vasko_kar@uctm.edu (V.K.); martin@uctm.edu (M.B.); 2 Institute of Electrochemistry and Energy Systems, Bulgarian Academy of Sciences, 1113 Sofia, Bulgaria

**Keywords:** Fe_3_O_4_ {111} plane, small molecules, atomistic MD, Large-scale Atomic/Molecular Massively Parallel Simulator (LAMMPS), Clay FF

## Abstract

The present study proposes an atomistic molecular dynamics model system of a magnetite (Fe_3_O_4_) {111} surface. The effect of temperature on the adsorption process of ammonia (NH_3_) at low concentrations in the aqueous phase has been considered. The molecular dynamics simulations were carried out using the Clay force field (Clay FF) with a modification for the iron atoms in the NPT ensemble at a pressure of 90 bar. The considered system was heated in a temperature range from 293 to 473 K, and additional relaxations were performed at temperatures of interest. Within the scope of this study, the basic parameters of the magnetite surface were calculated and the distances between the ammonia molecules and the surface were determined. A general idea of the degree and rate of adsorption at specific temperatures was obtained. The calculation results were compared to the experimental data where possible and to other available simulations of adsorption processes on metal oxides.

## 1. Introduction

Steam generators are key components of nuclear power plants, as their reliability affects the overall operation of the plant. A common operational problem that occurs in steam generators is the corrosion of the structural material and the build-up and deposition of unwanted solids on its internal surfaces. Steel is chosen for most applications because of its properties such as strength, durability, ease of fabrication and cost. Iron alloys have the property of resisting corrosion by forming a thin layer of oxide [1]. The area of focus in this research is magnetite Fe_3_O_4_ that approximates the protective layer on low-carbon steel. Notwithstanding the fact that the real structure of such a layer could be amorphous, strongly clustered and with bound surface hydroxyls, understanding the characteristics, formation and transformation of Fe_3_O_4_ can contribute to a reduction in material damage and waste, as well as the development of new products with improved qualities for various applications. The crystal structure of magnetite follows an inverse spinel lattice with alternating octahedral and tetrahedral–octahedral layers [2]. A detailed atomistic understanding of magnetite in complex nanostructures and of surface interactions is essential for many fundamental and technological processes.

Ammonia (NH_3_) is one of the principal components of secondary circuit water chemistry [3] which ensures that the pH of the coolant remains sufficiently alkaline to minimize the uniform corrosion of steel. Recently, it has been reported that whereas at low concentrations (<10^−4^ mol dm^−3^) it acts as a blocking inhibitor on carbon steel, at high concentrations (>10^−3^ mol dm^−3^), inhibiting ammonia is removed via the formation of an ammoniated Fe(II) complex and the surface is activated [4]. In recent years, the interaction of ammonia with various iron oxides was studied in the context of using its catalytic oxidation as a promising method for its removal from the atmosphere [5,6,7,8]. The adsorption behavior of NH_4_^+^ on a magnetite–montmorillonite composite was well expressed by pseudo-second-order kinetics and a Temkin isotherm model [5]. On γ-Fe_2_O_3_, NH_3_ was preferably adsorbed on the octahedral Fe site, with the N atom bonding to that site [7]. A Mars−van Krevelen-type surface NH_3_ oxidation mechanism was proposed for Fe oxide supported on Al_2_O_3_: the lattice oxygen of Fe oxide was used to oxidize adsorbed NH_3_ to −NH_2_, which dimerized into N_2_H_4_ intermediate species and was then desorbed as N_2_ [8].

An accurate description of iron oxide/water electrostatic interactions depends on the correct selection of a force field (FF) and the parameters used to describe the bond between surface iron and oxygen atoms from water molecules. It is still challenging to model magnetite with empirical force fields using fixed point charges due to the lack of accurate approximations of the electronic structure and the complex oxidation state of magnetite. Using volume charge values for surface atoms results in an “unphysical” surface [9,10,11]. An accurate approximation of the charge distribution is necessary to obtain realistic surface structures and to model the correct mechanical behavior of magnetite. Thanks to advances in computational algorithms and the accuracy of empirical force fields, atomistic-scale simulations have now provided a deeper insight into many other iron oxide-based materials that are not magnetite-based [12,13]. Studying oxidations from an atomistic point of view can offer new insights into the behavior of atoms and the influencing factors in oxidation mechanisms [14]. The number of atomistic molecular dynamics models of a magnetite surface is rather small, given the difficulties in describing the spin distribution. Also, the possibilities of adsorption on this type of surface are not sufficiently well studied.

The Clay FF [15] is a force field suitable for the simulation of hydrated and multicomponent mineral systems and their surfaces with aqueous solutions. The approach in this field is to treat most interatomic interactions as nonbonding. This allows the use of the force field for a wide variety of phases and correctly accounts for energy and momentum transfer between the liquid phase and the solid, while keeping the number of parameters small enough to allow the modeling of relatively large systems. Simulating bulk magnetite requires adapting the parameters for the short-range repulsive interactions that represent the hematite–water interatomic potentials. There are a number of developments that propose parameters for bulk magnetite and its surface [15,16,17]. In the present work, another method was applied [18] to determine the iron atoms with octahedra and tetrahedra without separating them according to their place in the crystal (in the volume or on the surface). In the transferable intermolecular potential with 3 points (TIP3P) solid water model [19], the molecule is divided into three force centers corresponding to the three atoms of the water molecule and is implemented in the Clay FF [15].

The main goal of the present work is the construction of a model surface of magnetite Fe_3_O_4_, considering the {111} plane. It is essential to track the basic characteristics of the systems within the simulation time. Another important part of this study is the characterization of the behavior of ammonia molecules placed in an aqueous phase near the magnetite surface. The possibility of adsorption of these molecules on the surface of magnetite was evaluated.

## 2. Results and Discussion

From the atomistic molecular dynamic simulations of the system performed at the investigated temperatures, the trajectories with the coordinates and velocities of each atom were taken. The basic parameters related to the adequacy of the composite structure of magnetite under the imposed conditions were determined, information was obtained on the interaction of water molecules with the surface of the oxide, and the initial movement of some of the NH_3_ molecules was described. The radial distribution functions between the atoms involved in the interactions were calculated. The data presented were averaged from parallel simulations performed for each temperature. Also, the analyses were averaged by the number of particles and by time for the entire length of the trajectory or for a given period. The obtained results were compared with similar simulations and the experimental data.

### 2.1. Layer Properties

**Figure 1:**
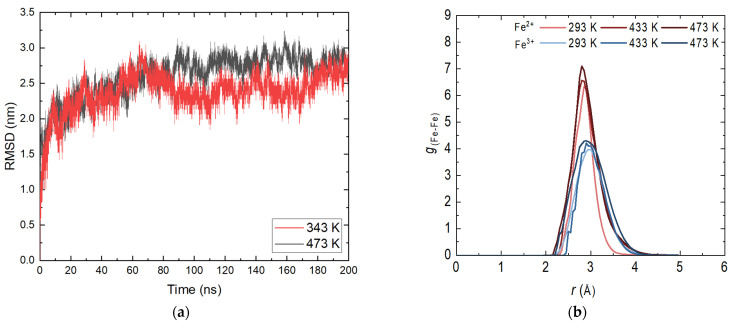
(**a**) Root mean square deviation (RMSD) of the magnetite system in the aqueous phase; (**b**) Radial distribution functions (RDFs) of the system magnetite in the aqueous phase.

A main criterion for the correctness of the simulations and the behavior of the magnetite layer is the root mean square deviation (RMSD) profile. Figure 1a shows the profiles at 343 and 473 K. For the other investigated temperatures, the shape of the curve is analogous with small differences in intensity.

At the beginning of the display period, there is a slight increase in the values characteristic of the relaxation system. A reduction in fluctuations is also observed, again related to the stabilization of the system. There is no distinct transition or distinct fragments, indicating that no energy transition is observed in the layer. This is an expected result given the periodic structure of the nanolayer and the significantly strong bonds within it. As expected, there are no clearly defined substructures at different temperatures. A slight increase in the movement of atoms with increasing temperature is observed, which does not affect the stability of the layer. The presence of dissolved molecules in the aqueous phase does not affect the overall RMSD profile given the length of the trajectories. These results are completely satisfactory and enable the continuation of the atomistic molecular dynamics simulations with the confirmed geometries of the demonstration systems.

In order to determine possible deformations in the structure of the crystal system, it is appropriate to consider the radial distribution functions (RDFs). The determination was made for both types of iron ions by obtaining the most probable distance between iron atoms with the same charge. Figure 1b shows the RDF profile for the productive part of the 50 ns long simulation before adding ammonia to the aqueous phase. Statistical analysis was conducted on the last 10 ns. The distributions of Fe^2+^ and Fe^3+^ are typical of a close-range ordered structure. There is no distinct second or third peak, indicating that at this stage the structure remains unchanged within the simulation time. Like the RMSD curves, there is no significant difference in shape with respect to temperature, and again there is a slight increase in peak intensity and width. An exchange of iron atoms is possible [20] with the tetrahedral sites being more occupied by Fe^2+^ [21]. The preferred distance between the iron atoms differs slightly from that of an ideal crystal [22]. Due to differences in the imposed temperature and pressure, the results obtained are in good compliance with the applied methodology [18]. From the reasoning made, it is clear that the constructed layer of magnetite is adequate; there is no interaction of the bulk crystal with water and the system can be used in combination with ammonia.

### 2.2. Surface Interaction

**Figure 2:**
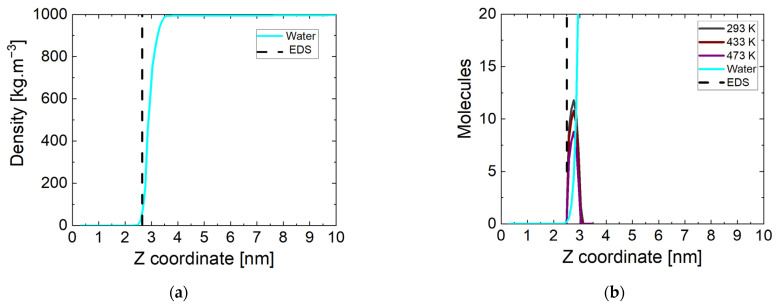
(**a**) Water mass density profiles at the beginning of the simulation time, EDS: equimolecular dividing surface; (**b**) number of water and ammonia molecules near the magnetite at the end of the simulation time at three temperatures.

A more accurate picture of the state of water and surface interactions can be made by calculating the mass density profile of water molecules relative to the z direction in the periodic box. The simulation box is divided into sub-volumes (20) in the direction parallel to the Fe_3_O_4_ magnetite surface. In these sub-volumes, the atoms of the corresponding type of molecule are counted and referred to the entire volume. The end result is a mass density profile for a given component of the investigated systems in the periodic box. The data for the considered surface at the beginning of the simulation period are shown in Figure 2a at 298 K. All simulations started from the same initial coordinates of the atoms and accordingly there are no differences in the state of the system at the initial stage regarding the surface. The dashed line corresponds to the boundary surface of magnetite determined at a solid phase density of 0 kg m^−3^. The correct density of water in the bulk phase is reached and there is no intrusion of molecules into the crystal. At this stage of the simulations, there is no pronounced increase in the mass density of the water phase near the surface.

The atoms in the same sub-volumes at the end of the simulation were counted in the manner described above. The results are shown in Figure 2b, but showing the number of molecules rather than the density. Here, the equimolecular dividing surface (EDS) determined at a water phase density of 500 kg m^−3^ is represented by a dashed line. The indicative data are at a temperature of 293 K, and there are no significant differences in the other values. In all systems near the surface of the magnetite, there is a peak formed corresponding to the ammonia molecules approaching its surface. Their number is the same (~11) at all temperatures except 433 K and 473 K. At lower temperatures, only one non-adsorbed molecule remains. One of the possible reasons for this is the too-large distance to the surface of the oxide, and another is the cut of the nitrogen atom in the molecule. At the two higher temperatures, fewer molecules (respectively, ~10 and ~8) reach the surface in all four runs. The attraction of ammonia molecules to the magnetite surface is unambiguous, due to the distribution of surface charges on the iron atoms. The number of molecules approaching the surface is largely influenced by the state of the water phase and the ability of the particles to move.

**Figure 3:**
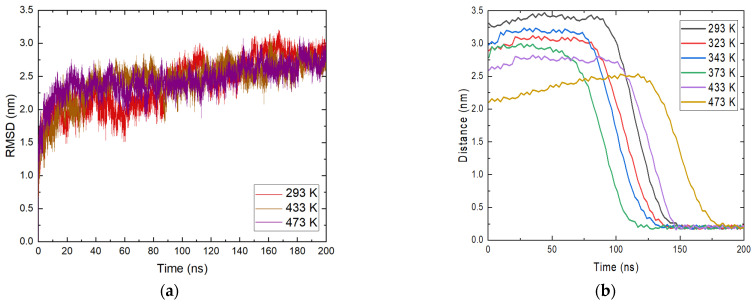
(**a**) Root mean square deviation (RMSD) of ammonia molecules at different temperatures; (**b**) position of ammonia molecules relative to the magnetite surface at all studied temperatures.

This necessitates a more careful study of the reported phenomenon. The RMSD profile of ammonia was calculated and the position of molecules relative to magnetite within the entire trajectory was determined. The data are illustrated in Figure 3a.

An analysis for NH_3_ was carried out for all molecules placed in the system. The reason for this is the clear approach to the magnetite surface of most ammonia molecules. The profile in Figure 3a shows temperatures of 293 K, 433 K, and 473 K. Similar to the analyses made so far, the general behavior of ammonia does not seem to vary significantly with temperature. From the RMSD plot, at 293 K, a change in motion, or more precisely a change in configuration, is observed between 100 and 140 ns. At 473 K, there is a change in their profile of 180 ns, which also corresponds to the moment of adsorption of ammonia molecules. At the end of the simulation time for all systems, there is a decrease in fluctuations, indicating that there is no transition in the structures. This indicates that there is a change in the orientation of ammonia, which remains relatively constant within the simulations. Again, there is an increase in intensity with time and the fluctuations generally subside because of the relaxation of the system.

**Figure 4:**
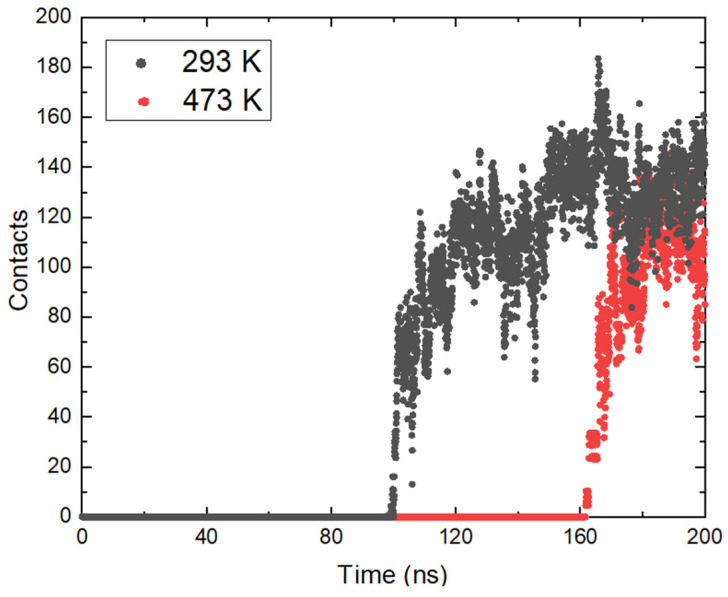
Number of contacts between ammonia molecules and the magnetite surface at two of the investigated temperatures.

The approach of ammonia NH_3_ was also tracked by determining the minimum distances between the center of mass of the molecules and the position of the magnetite surface relative to the z axis in the periodic box. The resulting distances are presented in Figure 3b. From the calculated trajectories, small distances between the surface and the considered ammonia molecules are estimated, which is a good indication of the contact between them, and it is possible that adsorption occurred. It is once again important to note that the initial coordinates at all temperatures are the same, but after heating in the NVT ensemble to the corresponding temperature there are differences in the position of the ammonia molecules. This is also due to the different distances of the ammonia molecules from the surface at the beginning of the trajectories. At all temperatures, ammonia reaches the magnetite. Particles within the simulation time remain on the surface and this is a clear indication of adsorption. The approach of ammonia is affected by the temperature and with growth this phenomenon takes place at an earlier stage. The exception is the system at the highest temperature (Figure 3b). There, the average distance between ammonia and magnetite is smaller, but a sharp approach occurs after 150 ns. In this system, the ammonia molecules that reached the surface are fewer, in agreement with the analysis presented in Figure 2b. The considered profiles show a clear tendency for the NH3 molecules to come closer together and the desire to stay permanently in the surface area. Additional information about ammonia adsorption can be obtained from the number of contacts of the molecule with the surface within the simulation. The calculated data are shown in Figure 4. The numbers of contacts between ammonia molecules and the magnetite surface at two of the investigated temperatures are shown in Figure 4.

The data show a relatively rapid increase in the number of contacts as the molecules approach the oxide. Although the average distance at the highest temperature (473 K) increases more slowly compared to the others, after a certain time (160 ns) it rises sharply. In general, the total number of contacts is smaller compared to 293 K, which is in good agreement with the smaller number of adsorbed molecules. The time taken for the number of contacts to grow at both temperatures is relatively the same. At 293 K, an increase from single contacts to over 100 is reached in 14.6 ns; at 473 K, the contact development period is 16.8 ns. This shows that after the ammonia molecules reach a certain distance from the surface, the effect of temperature is not so significant, and the adsorption depends on the electrostatic interaction between the atoms.

### 2.3. Radial Distribution Functions

**Figure 5:**
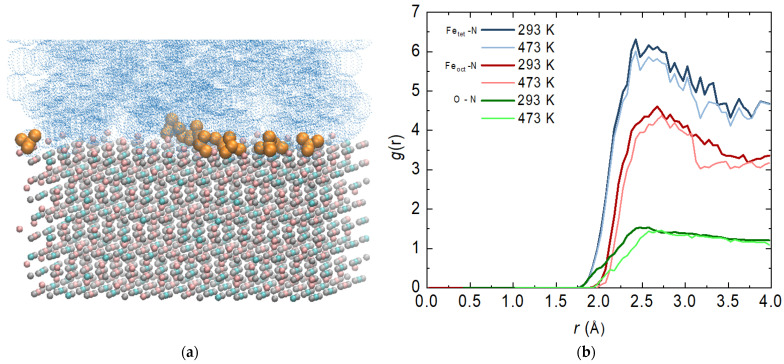
(**a**) Final configurations of the system composed of magnetite Fe_3_O_4_ (Fe^3+^ in pink; Fe^2+^ in cyan; O^2−^ in grey), aqueous phase (in blue) and ammonia molecules (in dark yellow) at 293 K; (**b**) radial distribution functions (RDFs) of the atoms from magnetite and the nitrogen atom from ammonia—bold lines are at 293 and regular lines are at 473 K.

It is important to mention that with surface phenomena in general, it is of particular importance with which functional group of the molecule the surface is attacked. At this stage, visual observations show that most of the ammonia reacts with its nitrogen atom (Figure 5a). A similar phenomenon has been observed for a single molecule of hydrazine adsorbed on an Fe(110) surface [23]. This necessitates a more detailed examination of the interactions between specific atoms.

Based on this, the most probable distance between the nitrogen atom of the ammonia molecule and the iron atoms of the two types of tetrahedral and octahedral sites (regardless of the charge) was calculated. The distance to the oxygen atoms was also determined. The function profiles overlap at different temperatures, which is not a completely expected result (Figure 5b). With an increase in temperature, there is a slight decrease in the intensity of the peaks, but there is no shift in their place. Once they have reached the surface of the magnetite, the attraction of the molecules is more influenced by the charge of the atoms than the current by the temperature. Both types of magnetite target atoms show strong fluctuations related to the possible movement of ammonia molecules on the surface. One cannot speak of an adsorbed layer, but the presence of a peak is a clear indication of such a phenomenon. Tetrahedral sites are largely preferred, as there is a distribution of both charges. The interaction with the iron atoms in the octahedral positions is also significant, but not as intense. The interaction with oxygen atoms from the magnetite surface is weakest.

**Figure 6:**
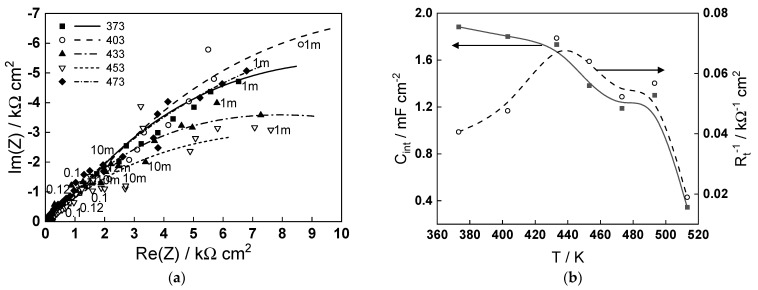
(**a**) Electrochemical impedance spectra of carbon steel in an ammonia solution (pH 9.8) at the free corrosion potential in the temperature interval 373–473 K (parameter is frequency in Hz); (**b**) temperature dependence of the interfacial capacitance (C_int_) and the inverse of the charge transfer resistance (R_t_^−1^) estimated from fitting the impedance spectra to the transfer function of the Mixed-Conduction Model.

To put the obtained modeling results into perspective in relation to the corrosion of carbon steel in steam generator coolant, the impedance spectra measured after 70 h of exposure in a de-oxygenated ammonia solution (pH 9.8 at room temperature) at temperatures in the range of 373–473 K are presented in Figure 6a. As already reported for similar steel in ammonia–ethanolamine (AMETA) coolant [24], the low-frequency limit of the impedance that can be correlated with the inverse of the corrosion rate behaves non-monotonously with temperature, exhibiting a minimum at 160–180 °C. The complete interpretation of the spectra using the Mixed-Conduction Model for Oxide films [25] will be reported in a companion paper. Here, we focus our attention on the parameters characterizing the magnetite (oxide formed on steel)/coolant (ammonia + water) interface—the interfacial capacitance (C_int_) and charge transfer resistance (R_t_). The temperature dependence of these parameters is illustrated in Figure 6b. The order of values of C_int_ (0.4–2.0 mF cm^−2^) is typical for an adsorption pseudo-capacitance at the oxide/water interface and is relatively constant in the interval 373–433 K, indicating no change in the adsorption mechanism. At higher temperatures, the capacitance decreases, suggesting a mode compact adsorption layer or a smaller coverage of the adsorbed species, which is in line with the modeling findings of an alteration of ammonia adsorption when the temperature approaches 473 K. The maximum of the inverse of the charge transfer resistance at 433–453 K, indicating the maximum rate of the cathodic coupled reaction of corrosion (water reduction), can also be tentatively ascribed to the modification of the adsorption layer leading to an alteration of the mechanism of water reduction. Further investigations are in progress to elucidate the influence of ammonia on the thickness and composition of the oxide and corrosion release rates of carbon steel in simulated steam generator conditions.

## 3. Systems and Methods

Magnetite has a spinel structure and crystallizes in the cubic space group Fd̅3m. In the present study, a structure at high pressures [26] was used for the unit cell, keeping all the basic parameters of the crystal lattice. A detailed description of the structure and mode of replication of the unit cell is given below.

The system contained a total of 56 atoms with a side length of 8.44 Å, which was the lattice constant of 16 Fe^3+^ atoms, 8 Fe^2+^ atoms and 32 O^2−^ atoms. The Fe^2+^ atoms were bonded to four equivalent O^2−^ atoms to form FeO_4_ tetrahedra, which shared angles with twelve equivalent FeO_6_ octahedra, the angles being 57°. All Fe^2^⁺/O bond lengths were 1.92 Å. The Fe³⁺ atoms were bonded to six equivalent O^2−^ atoms to form FeO_6_ octahedra, which shared corners with six equivalent FeO_4_ tetrahedra and edges with six equivalent FeO_6_ octahedra. All Fe^3+^/O bond lengths were 2.06 Å. The O^2−^ atoms were bonded in a distorted rectangular cradle-like geometry to one Fe²⁺ and three equivalent Fe^3^⁺ atoms. A major challenge in modeling was the clear distinction between the charges of the iron atoms and their spatial orientation.

**Figure 7:**
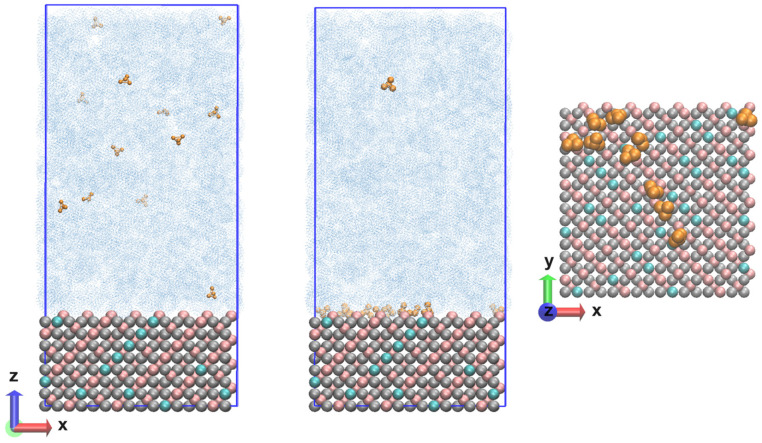
Nanolayer composed of magnetite (Fe^3+^ in pink; Fe^2+^ in cyan; O^2−^ in grey) and an aqueous phase (in blue) with ammonia molecules (in dark yellow) at the beginning (**left**) and end (**center**) of the calculated trajectories and the surface in the normal direction (**right**).

The resulting Fe_3_O_4_ unit cell structure was multiplied in the three directions in such a way as to reproduce the {111} plane. The number of elementary units was 48 and the system contained a total of 2688 atoms. The size of the layer along the x and y axis is 3.39 nm and the thickness was 2.50 nm. With the layer thus constructed, it was possible to perform atomistic molecular dynamics simulations with the Clay force field. Simulating bulk magnetite requires adapting the parameters for the short-range repulsive interactions that represent the hematite–water interatomic potentials [18]. It was proposed to divide the atoms into octahedral and tetrahedral atoms only, with the partial charges being distributed equally for the entire system. It turned out to be a reasonable choice to choose the opposite partial atomic charge of O^2−^ for Fe^2+^, as this provided a neutral charge on the bulk magnetite unit cell. Moreover, using this partial atomic charge for Fe^2+^ led to a good description of bulk magnetite, and of magnetite surfaces. The approach presented here ultimately allowed the simulation of a realistic and much larger system. It is also suitable for studying the mechanical and structural properties of functionalized magnetite surfaces and nanoparticles. This opens the door to a more fundamental understanding of the oxidation state ordering of inverse spinel structures. As can be seen, understanding the chemical activity of Fe_3_O_4_ is challenging due to the complex chemical behavior of magnetite on the surface. In the present study, the Clay FF was applied with charge and bond modifications [18]. Periodic boundary conditions (PBCs) with changed dimensions along the z coordinate of 10 nm were imposed on the already built layer of Fe_3_O_4_. In this empty volume, there were 12 ammonia molecules, and the concentration reached was 1.8 10^−4^ mol dm^−3^. In the next step, the model system was saturated with water molecules until the correct density was reached for the given conditions. The number of water molecules was 3437, so the atoms in the system exceeded 13,000. The thickness of the water layer was large enough that there was no interaction with the periodic image. The initial coordinates of the system are shown in Figure 7 (left).

The calculations were performed with the Clay force field [15], which was chosen as the most suitable for describing periodic structures and at the same time containing parameters for different functional groups for possible solvents. A leap-frog integrator of the equations of motion [27] was used in combination with the PME (Particle mesh Ewald) method for the electrostatic interactions [28] at a switched cut-off of 1.2/1.4 nm. The same cut-off was imposed for the van der Waals interactions, which were evaluated with the Lennard-Jones potential. The water model was TIP3P [29]. All simulations were performed in the NPT ensemble at several temperatures (293 K; 323 K; 343 K; 373 K; 473 K) and a pressure of 90 bar was held constant with a Nose–Hoover’s thermostat [30,31] and a Parrinello–Rahman’s barostat [32,33]. NPT pressure scaling was applied—dependent on all coordinates (x,y,z) for all models in PBCs. The behavior of water at different temperatures was tailored to the viscosity according to damp = m/(3 × pi × μ × d), where the dynamic viscosity of water varies with respect to the temperature [34]. The systems underwent energy minimization, heating and relaxation, the latter being verified by reaching a constant average of the total energy, temperature and pressure. The root mean square deviation of the coordinates of the two types of iron ions and the dissolved molecules in the aqueous phase was also monitored. The trajectory length was 200 ns, with some results presented for the last 50 ns. The time step was ∆t = 0.5 fs, and each frame in the trajectories was saved for 10 ps. For each temperature, four independent runs were made with different configurations for the ammonia and water molecules and with different velocities for the magnetite atoms, but the same initial coordinates in the model system. In order to follow the evolution of the systems in more detail, some of the statistical analyses were made for simulation intervals of 10 ns. Molecular dynamics (MD) calculations were performed with the LAMMPS 2Aug2023 (Large-scale Atomic/Molecular Massively Parallel Simulator) software package with a focus on the modeling of inorganic materials [35,36]. A demo version of the source script is attached in the Supplementary Material. The program VMD 1.9.4a (Visual Molecular Dynamics) [37] was used to visualize the results. The final coordinates (state) of the system are shown in Figure 7 (center) with a top view of the surface with ammonia molecules near the magnetite Figure 7 (right).

The electrochemical impedance spectra of low-carbon steel (22 K type) were measured in an NH_3_ solution (pH 9.8) at the corrosion potential in the temperature interval 373–513 K and a pressure of 90 bar in a high-temperature re-circulation loop using a Compactstat 10030 potentiostat equipped with a frequency response analyzer module (Ivium, Eindhoven, The Netherlands). The spectra were measured in a frequency range of 0.5 mHz to 11 kHz with an ac signal of 40 mV (rms).

## 4. Conclusions

The main goal of the present work was the construction of a model system resembling the {111} plane of magnetite Fe_3_O_4_. The construction of this surface is at the molecular level, for which the methods of molecular mechanics were used. Given the spinel structure of this crystal, there are a number of difficulties in the selection of a force field and its modifications. For the present study, the Clay FF, also used in thee molecular dynamics simulations of a large number of long-range structures, was imposed. Atomistic systems containing magnetite, aqueous phase and ammonia molecules at different temperatures were built with the selected parameters and simulations using molecular dynamics were carried out. Statistical analyses were performed on the obtained trajectories, proving the stability of the developed systems. The root mean square deviation profile (RMSD), radial distribution functions (RDFs) and mass density profile for the aqueous phase were determined, and the movement of ammonia was analyzed. The constructed model structures are stable and there is no clearly pronounced energy transition in the systems. There is a definite approach of part of the dissolved ammonia molecules to the magnetite surface. The time for the observed phenomenon depends on the temperature, but not the interaction itself. The influence of temperature on the extent of the adsorption of ammonia is tentatively correlated with the interfacial parameters of the electrochemical impedance spectra of carbon steel measured in an ammonia–water solution in a similar temperature interval.

## Data Availability

The data presented in this study are available on request from the corresponding author due to privacy.

## Supplementary Materials

The following supporting information can be downloaded at: https://www.mdpi.com/article/10.3390/molecules29143276/s1. LAMMPS input demo script for bulk magnetite.
